# Methods of spatial cluster detection in rare childhood cancers: Benchmarking data and results from a simulation study on nephroblastoma

**DOI:** 10.1016/j.dib.2020.106683

**Published:** 2020-12-29

**Authors:** Michael M. Schündeln, Toni Lange, Maximilian Knoll, Claudia Spix, Hermann Brenner, Kayvan Bozorgmehr, Christian Stock

**Affiliations:** a Pediatric Hematology and Oncology, Department of Pediatrics III, University Hospital Essen and the University of Duisburg-Essen, Essen, Germany; b Center for Evidence-based Healthcare, University Hospital and Faculty of Medicine Carl Gustav Carus, TU Dresden, Germany; c Clinical Cooperation Unit Radiation Oncology, German Cancer Research Center (DKFZ), Heidelberg, Germany; d German Childhood Cancer Registry, Institute for Medical Biostatistics, Epidemiology and Informatics (IMBEI), University Medical Centre of the Johannes Gutenberg University Mainz, Mainz, Germany; e Division of Clinical Epidemiology and Aging Research, German Cancer Research Center (DKFZ), Heidelberg, Germany; f Division of Preventive Oncology, German Cancer Research Center (DKFZ) and National Center for Tumor Diseases (NCT), Heidelberg, Germany; g German Cancer Consortium (DKTK), German Cancer Research Center (DKFZ), Heidelberg, Germany; h Department of Population Medicine and Health Services Research, School of Public Health, Bielefeld University, Bielefeld, Germany; i Institute of Medical Biometry and Informatics (IMBI), University of Heidelberg, Heidelberg, Germany

**Keywords:** Simulation study, Random distribution, Spatial cluster, Childhood cancer, Nephroblastoma, Spatial scan statistic, Besag-Newell, Bayesian, Besag York Mollié

## Abstract

The potential existence of spatial clusters in childhood cancer incidence is a debated topic. Identification of rare disease clusters in general may help to better understand disease etiology and develop preventive strategies against such entities.

The incidence of newly diagnosed childhood malignancies under 15 years of age is 140/1,000,000. In this context, the subgroup of nephroblastoma represents an extremely rare entity with an annual incidence of 7/1,000,000. We evaluated widely used statistical approaches for spatial cluster detection in childhood cancer (Ref. Schündeln et al., 2021, *Cancer Epidemiology*). For the simulation study, random high risk clusters of 1 to 50 adjacent districts (NUTS-level 3, nomenclature des unités territoriales statistiques) were generated on the basis of the 402 German administrative districts. Each cluster was simulated with different relative risk levels (1 to 100). For each combination of cluster size and risk level 2000 iterations were performed. Simulated data was then analyzed by three local clustering tests: Besag-Newell method, spatial scan statistic and the Bayesian Besag-York-Mollié approach (fit by Integrated Nested Laplace Approximation). The performance characteristics of all three methods were systematically documented (sensitivity, specificity, positive/negative predictive values, exact- and minimum power, correct classification, positive/negative diagnostic likelihood and false positive/negative rate).

This data article links to a Mendeley online repository which includes the raw data of simulated high-risk clusters and simulated cases on the district level for an all-childhood-malignancy scenario as well as for cases of nephroblastoma. These data was used for the evaluation of the three cluster detection methods. The R code for simulation and analysis are available from GitHub.

The article also includes analyzed data summarizing the performance of the cluster detection tests in very rare disease entities, using the example of simulated nephroblastoma cases.

The raw data from the study can be used for benchmarking analyses applying different spatial statistical methods systematically and evaluating their performance characteristics comparatively. The analyzed data from the nephroblastoma example can be useful to interpret the performance of the three applied local cluster detection tests in the setting of extremely rare disease entities. As a practical application, data and R code can be used for performance analyses when planning to establish surveillance systems for rare disease entities.

**Abbreviations**
BN: Besag-Newell
BYM: Bayesian Besag-York-Mollié
CC: Correct classification
CP: Correct proportion
EP: Exact power
FNR: False negative rate
FPR: False positive rate
INLA: Integrated Nested Laplace Approximation
MCB: Monte Carlo Bias
MP: Minimum power
NDL: Negative diagnostic likelihood
NPV: Negative predictive value
NUTS: Nomenclature des unités territoriales statistiques
PDL: Positive diagnostic likelihood
PPV: Positive predictive value
Sens: Sensitivity
Spec: Specificity
SSS: Spatial scan statistics

      

**Specifications Table**

**Table:** 

Subject	Epidemiology
Specific subject area	Cancer Epidemiology, Childhood Cancer, Detection of Spatial Clusters, Statistical Epidemiology
Type of data	Downloadable Table: Complete analyzed results from simulation study Downloadable RData files: Complete raw data of simulation Table: Summary of analyzed results from simulation study Graph: Summary of analyzed results from simulation study
How data were acquired	Baseline data: Database of Global Administrative Areas, GADM, Version 3.6. [1] German Feral Statistical Office (German population data) [2] Simulated data: R environment for statistical computing, version 3.5.3 [3] RStudio platform, version 1.1.456 [4] R package SpatialEpi, version 1.2.3 [5] R package R-INLA, version 18.07.12 [6] Computational implementation of the simulation study are provided online at https://github.com/Pediatrics/Childhood-Cancer-Study
Data format	Raw data: RData file Analyzed data: Excel file
Parameters for data collection	High-risk clusters of defined size (1 to 50 adjacent districts) were randomly assembled on the district level in Germany. At baseline relative risk of 1 (RR=1), the incidence of nephroblastoma was set as 7/1,000,000 for all pediatric cancer cases as 140/1,000,000 [7]. Each high-risk cluster was simulated with 10 different RR-levels (1 to 100). For each combination 2000 iterations were done.
Description of data collection	Simulated raw data, consisting of randomly assembled clusters and simulated cases, was stored in RData files. Subsequently the simulated data was analyzed by Besag-Newell method, spatial scan statistic and Bayesian Besag-York-Mollié approach fit by Integrated Nested Laplace Approximation.
Data source location	Pediatric Hematology and Oncology Department of Pediatrics III University of Duisburg-Essen Essen, Germany
Data accessibility	1 Complete raw data of the simulation in the Mendeley repository: Schündeln, Michael (2020), “Childhood Cancer Cluster Simulation”, Mendeley Data, V4, https://data.mendeley.com/datasets/3hrg9tpsx9/4 2 Summary of analyzed data with the article 3 Complete analyzed data in the Mendeley Repository (see above)
Related research article	M.M. Schündeln, T. Lange, M. Knoll, C. Spix, H. Brenner, K. Bozorgmehr, C. Stock, Statistical Methods for Spatial Cluster Detection in Childhood Cancer Incidence: A Simulation Study, Cancer Epidemiol. 2020.

## Value of the Data

• The raw data from this study can be used for benchmarking analyses when applying other statistical methods and evaluating their performance characteristics systematically.
• The data is of benefit for researchers investigating the spatial epidemiology of extremely rare disease entities.
• The analyzed data from the nephroblastoma example can be useful to interpret the comparative performance of local cluster detection tests in the setting of extremely rare disease entities.
• Data and R code can be used for performance analyses when planning to establish surveillance systems for various disease entities.

## Data Description

### Raw data

The aim of the study was to evaluate three local clustering tests: Besag Newell (BN), spatial scan statistics (*SSS*) and the Bayesian Besag-York-Mollié approach (fit by Integrated Nested Laplace Approximation). To measure their performance, the tests were conducted with simulated data: Randomly assembled high-risk clusters of adjacent districts, increasing in size (*Cluster*) and in various risk levels (*RR*) were generated. The simulation process is described in detail in paragraph 2.3.

The raw data, generated by the simulation is presented online in the Mendeley repository (https://data.mendeley.com/datasets/3hrg9tpsx9/4). In the online repository, the raw data for the nephroblastoma incidence simulation is documented in the file "NephroblastomaSimulation.RData". The file for the simulation of the all-childhood-malignancies scenario is in the file "AllMalignancies.RData". Both files can be loaded into the statistical software R. Each file contains six lists for the different cluster sizes ("Cluster Size X"). Within each of these lists 2000 simulations for clusters in 10 different risk levels ("RR Y Cluster"). Corresponding to each run of simulation, the simulated cases for each of the respective scenario ("RR Y SimCases") are found. The files also contain the population of children under 15 years for each district (“District Population”) as published by the German Federal Statistical Office[2]. In addition the expected cases for the entities, all malignancies or nephroblastoma, (“Expected Cases”) per district, based on the expected incidence rates are given within the files.

The adjacency matrix for the 402 German districts is added as a separate RData file (Adjacency Matrix.RData).

### Analyzed data from Nephroblastoma example

In the study, the performance of the three spatial cluster detection tests was systematically documented (details see 2.5). The data in Table 1 summarizes the analyzed results using the example of nephroblastoma cases. Selected performance measures are displayed as percentage sensitivity (*Sens*), specificity (*Spec*), positive predictive value (PPV), negative predictive value (*NPV*), exact power (*EP*), minimum power (*MP*) and correct classification (*CC*).

**Table 1:** Random clusters of nephroblastoma. Selected performance measures.

		Besag Newell							SSS							BYM (INLA)						
Cluster	RR	Sens	Spec	PPV	NPV	EP	MP	CC	Sens	Spec	PPV	NPV	EP	MP	CC	Sens	Spec	PPV	NPV	EP	MP	CC
2	1	0.9	99.0	0.7	99.2	0.1	2.1	98.2	3.0	97.5	0.8	99.2	0.0	4.2	96.7	0.0	100.0	0.1	99.2	0.0	0.1	99.2
2	1.2	1.9	99.0	1.5	99.2	0.1	4.6	98.2	4.8	97.5	1.9	99.2	0.2	7.2	96.7	0.0	100.0	0.1	99.2	0.0	0.1	99.2
2	1.5	6.1	99.0	4.3	99.2	0.1	13.9	98.3	10.5	97.5	5.2	99.3	0.3	15.1	96.8	0.2	100.0	0.5	99.2	0.0	0.6	99.2
2	2	17.9	99.0	12.1	99.3	0.2	36.5	98.3	25.0	97.7	16.4	99.4	1.6	34.0	97.2	1.4	100.0	3.9	99.2	0.0	4.1	99.2
2	5	85.5	98.4	34.3	99.9	1.2	98.8	98.3	83.5	99.4	70.2	99.9	19.1	97.0	99.2	31.0	99.9	37.4	99.4	11.2	41.3	99.4
2	10	99.1	97.7	29.3	100.0	0.5	100.0	97.7	97.2	99.5	70.6	100.0	26.6	100.0	99.5	87.2	99.7	73.4	99.9	31.7	92.7	99.6
2	100	100.0	97.8	32.1	100.0	0.9	100.0	97.8	100.0	99.4	67.0	100.0	24.2	100.0	99.4	100.0	99.3	56.9	100.0	6.5	100.0	99.3
5	1	0.9	99.0	1.4	98.5	0.0	4.1	97.5	3.0	97.5	1.4	98.5	0.0	5.4	96.0	0.0	100.0	0.1	98.5	0.0	0.1	98.4
5	1.2	2.2	99.0	3.3	98.5	0.0	9.4	97.5	5.6	97.5	3.4	98.5	0.0	9.4	96.0	0.1	100.0	0.1	98.5	0.0	0.2	98.4
5	1.5	6.6	99.0	9.2	98.5	0.0	25.0	97.6	16.4	97.5	12.0	98.7	0.2	26.1	96.3	0.2	100.0	0.8	98.5	0.0	1.0	98.4
5	2	19.9	99.0	24.2	98.7	0.0	59.4	97.8	42.0	98.0	33.8	99.1	1.8	57.5	97.1	1.6	100.0	6.6	98.5	0.0	7.0	98.5
5	5	86.6	98.2	47.6	99.8	0.4	100.0	98.1	91.7	99.4	75.8	99.9	13.4	99.8	99.2	61.3	99.7	61.7	99.4	7.9	74.8	99.1
5	10	99.4	97.4	40.9	100.0	0.4	100.0	97.4	98.5	99.4	76.6	100.0	19.4	100.0	99.4	97.8	99.2	70.6	100.0	12.4	99.7	99.2
5	100	100.0	97.1	39.8	100.0	1.1	100.0	97.2	100.0	99.3	73.7	100.0	17.3	100.0	99.3	100.0	98.8	59.8	100.0	1.7	100.0	98.8
20	1	0.9	99.0	4.6	94.9	0.0	12.5	94.0	2.9	97.5	5.6	94.9	0.0	11.8	92.6	0.0	100.0	0.1	94.9	0.0	0.2	94.9
20	1.2	2.2	99.1	11.7	94.9	0.0	27.6	94.1	10.1	97.6	16.5	95.3	0.0	28.0	93.1	0.1	100.0	0.3	94.9	0.0	0.5	94.9
20	1.5	6.1	99.1	27.4	95.1	0.0	57.3	94.3	34.8	97.8	44.4	96.6	0.0	64.5	94.6	1.1	100.0	5.3	94.9	0.0	5.9	94.9
20	2	18.5	99.1	53.8	95.7	0.0	92.4	95.0	69.6	98.2	72.8	98.4	0.1	96.4	96.8	15.8	99.8	35.2	95.6	0.1	38.6	95.5
20	5	84.0	98.1	72.4	99.1	0.1	100.0	97.4	92.9	98.3	77.5	99.6	0.1	100.0	98.0	97.9	98.8	82.6	99.9	2.1	100.0	98.8
20	10	98.8	96.5	62.5	99.9	0.0	100.0	96.7	98.8	98.0	75.5	99.9	0.7	100.0	98.1	99.9	98.5	78.7	100.0	0.6	100.0	98.5
20	100	100.0	95.2	55.4	100.0	0.0	100.0	95.5	100.0	97.5	70.9	100.0	0.6	100.0	97.6	100.0	98.2	76.2	100.0	0.1	100.0	98.3
50	1	0.9	99.0	12.1	87.3	0.0	28.4	86.6	2.7	97.5	13.9	87.4	0.0	22.2	85.5	0.0	100.0	0.0	87.4	0.0	0.1	87.3
50	1.2	2.0	99.1	27.2	87.5	0.0	52.7	86.9	11.2	98.3	39.7	88.5	0.0	52.5	87.3	0.4	99.9	2.3	87.4	0.0	2.9	87.4
50	1.5	5.3	99.3	54.2	87.9	0.0	87.8	87.4	33.4	98.6	77.1	91.2	0.0	94.1	90.4	13.9	99.6	47.3	89.0	0.0	51.3	88.7
50	2	14.9	99.4	79.3	89.0	0.0	100.0	88.7	58.3	98.3	84.2	94.3	0.0	100.0	93.2	64.3	98.7	89.3	95.1	0.0	98.9	94.4
50	5	73.0	98.4	87.0	96.2	0.0	100.0	95.2	90.1	97.8	85.8	98.6	0.0	100.0	96.8	98.7	97.7	86.7	99.8	0.3	100.0	97.9
50	10	95.4	96.6	80.8	99.3	0.0	100.0	96.4	97.2	97.7	86.1	99.6	0.0	100.0	97.6	99.9	97.7	86.4	100.0	0.1	100.0	98.0
50	100	100.0	93.6	70.4	100.0	0.0	100.0	94.4	100.0	97.5	85.9	100.0	0.0	100.0	97.8	100.0	97.9	87.7	100.0	0.2	100.0	98.2

The performance measures sensitivity (Sens), specificity (Spec), positive predictive value (PPV), negative predictive value (NPV), exact power (EP), minimum power (MP) and correct classification (CC) are displayed as percentage.

**Fig. 1:**
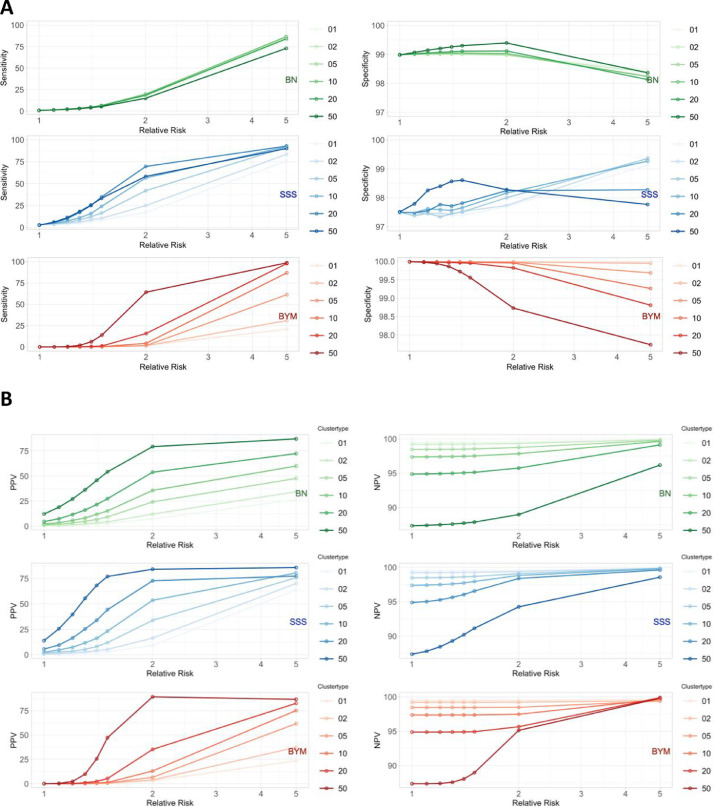
Performance, detecting random clusters of nephroblastoma. Sensitivity and specificity of Besag Newell (green), SSS (blue) and Besag York Mollié (INLA, red) method (%) as a function of relative risk. B) positive predictive value (PPV), negative predictive value (NPV) as a function of RR for three methods.

Fig. 1 gives an overview of results on sensitivity, specificity, PPV and NPV for each of the three methods separately, depending on relative risk and cluster size.

**Fig. 2:**
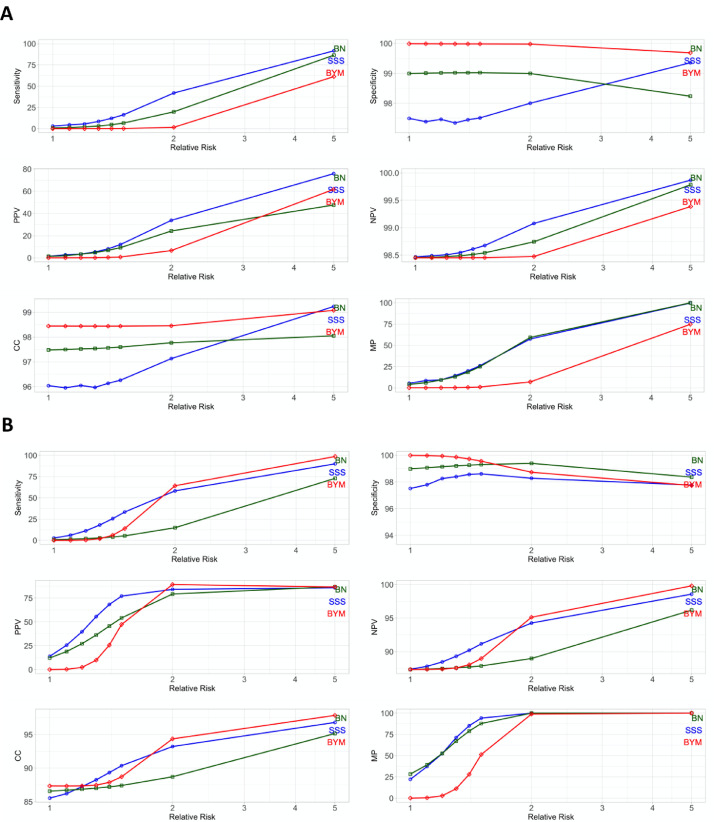
A) Detection of 5 random districts, B) Detection of 50 random districts. Sensitivity, specificity, positive predictive value (PPV), negative predictive value (NPV), correct classification (CC), minimum power (MP) in percent as a function.

The examples of a small and a large cluster high-risk scenario, 5- and 50-district clusters respectively, are shown in Fig. 2.

The complete analyzed data can be found in the Mendeley repository *(*(https://data.mendeley.com/datasets/3hrg9tpsx9/4, file “Analyzed Data.xlsx”). The complete data includes the results of the nephroblastoma scenario for all simulated RR levels and all simulated cluster sizes. In addition to the performance measures presented above, the correct proportion (CP), the positive diagnostic likelihood (*PDL*), negative diagnostic likelihood (*NDL*), false positive (*FPR*) and false negative rate (*FNR*) are displayed including the respective upper and lower confidence intervals.

Additionally, the file includes the complete analyzed data for the all-childhood-malignancies scenario (presented in detail in *Ref. Cancer Epidemiology*).

## Experimental Design, Materials and Methods

### General aspects of simulation study

For our study, we simulated random spatial clusters of the extremely rare childhood cancer subentity of nephroblastoma. The clusters varied in size and magnitude of risk increase. Overall incidence, population and spatial structure (districts) reflect conditions in Germany. The simulation was also performed for an all childhood malignancy scenario (see Ref. Schündeln et al., 2020, Cancer Epidemiology 2020). We systematically varied input parameters in the simulation and assessed performance of three cluster detection methods.

The code for the computational implementation of the simulation to reproduce the analysis of the published study is provided online (https://github.com/Pediatrics/Childhood-Cancer-Study). Here also the GADM shapefiles and the baseline population data can be found.

## Notation

Table 2 summarizes the notation for the explanatory remarks in the next paragraphs.

**Table 2:** Notation.

Variable	Definition
*RR_i_*	relative risk in district *i*
*c_i_*	number of cases in district *i*
*u_i_*	population size of district *i*
*e_i_*	expected number of cases in district *i*
*C*	total number of cases
*N*	total population size
*H*	total number of districts
*D_j(i)_*	total number of cases in district *i* and its *j* closest neighbors
*U_j(i)_*	population size in district *i* and its *j* closest neighbors

**Table 3:** Performance parameters.

Measured Parameter	Definition
Minimum Power (MP)	Proportion of simulations detecting at least one district of the true cluster
Exact Power (EP)	Proportion of simulations detecting the true cluster without false positives
Sensitivity (sens)	Proportion of correctly detected districts in the true cluster
Specificity (spec)	Percentage of normal risk districts, correctly classified as normal risk districts
Positive predictive value (PPV)	Proportion of districts in the detected cluster belonging to the true cluster
Negative predictive value (NPV)	Proportion of districts not labeled as a risk cluster that is not part of the true cluster
Correct classification (CC)	Percentage of correctly classified districts of all districts
Positive diagnostic likelihood (PDL)	The ratio of high-risk districts being detected, divided by the probability non-high-risk districts being detected (sensitivity / (1-specificity)
Negative diagnostic likelihood (NDL	The ratio of high-risk districts not being detected divided by the probability of non-high-risk districts not being detected ((1 – sensitivity) /specificity)
False positive rate (FPR)	Incorrectly labeled high-risk districts of all detected high-risk districts
False negative rate (FNR)	Incorrectly labeled normal-risk districts of all detected normal-risk districts
Monte Carlo Error (MCE) [20,21]	standard deviation of the Monte Carlo estimator (RR), taken across repetitions (n)

### Simulation and raw data generation

For the simulation and analysis of spatial data at the district-level (nomenclature des unités territoriales statistiques, NUTS-level 3 we used a shapefile obtained from the Database of Global Administrative Areas [1]. It represents 402 German districts according to the German administrative divisions of mid-2016. Corresponding population sizes, as of 31 December 2017, were obtained from the German Federal Statistical Office (Statistisches Bundesamt) [2]. The German pediatric population was estimated to be 11,048,523 children under the age of 15 years (13.3%). The number of children at risk below the age of 15 years for each spatial unit ranged between 3,594 and 492,448. The population density of children under 15 years of age ranged from 4 to 620 per km^2^.

High-risk clusters of different sizes were generated by randomly compiling a number of 1, 2, 3, 5, 10, 20 or 50 adjacent districts. A random district was (repeatedly) selected as a starting point using a fixed seed. Neighboring districts were identified using the adjacency matrix (evaluation of rows and columns) with recursion. The operation was terminated when the desired cluster size was reached. In case “donut-shaped” polygons were selected by the random process, the enclosed district was included into the generated risk cluster.

Crude incidence rates of expected pediatric cancer cases were assumed to follow a Poisson distribution with $\lambda_{nephroblastoma}=7/1\;000\;000$ (or $\lambda_{all}=140/1\;000\;000$ for the “all-pediatric-cancer-scenario”) [7,8]. Generally, *RR_i_* was assumed to be 1, while *RR_i_* associated with the generated clusters was varied in 10 steps from 1 to 100 (1, 1.1, 1.2, 1.3, 1.4, 1.5, 2.5, 10, 100), thus $\lambda_{\;i}\;=\;\lambda_{nephroblastomal}\times RR_{i}$. The data was aggregated over a 10-year period, as is regularly done for spatial epidemiological analysis of childhood cancer using population-based cancer registry data [7]. Therefore, the case numbers per district during the time period were calculated as follows: $c_{i,\;10}\;=\;\sum_{n=\;1}^{10}\lambda \;\times \;1\;000\;000\times u_{i,\;10}$. The crude incidence rates for each district were then calculated as follows: $cir_{i}\;=\;\frac{c_{i10}}{u_{i10}}\times \;1\;000\;000\;.$ Cancer incidence was simulated for all 402 districts 2000 times for each scenario. The simulation estimand was the district cumulative *RR (*$RR_{i}=\;\frac{\sum_{y=1}^{10}c_{i}}{\sum_{y=1}^{10}e_{i}}\;$).

### Cluster detection methods

#### Besag and Newell method

The first approach was introduced by and named after Besag and Newell [9]. Here a test for each single region *i* based on the number of neighbors that must be combined to contain a minimum number of user defined cases *k*. The cases surrounding district *i* are ranked according to their distance to *i* to identify the *k* nearest cases. The area containing those *k* nearest cases is then identified (*M_i_*), in which *M_i_* constitutes a possible disease cluster. The following explanatory remarks are based on Song and Kulldorff [10]. To test for clustering around *i*, the approach considers whether the total number of cases in *M_i_* is large relative to the total risk population. The test statistic is defined as follows: $R=\sum_{k=0}^{H\;}c_{i}I\left(P\left(M_{i}\leq m_{i}\;\right)<0.05\right)\;,$ where *M*_i_ is a random variable denoting the minimum number of districts needed to have at least *k* cases in district i and its *M*_i_ closest neighboring districts, *m_i_* is the observed value of *Mi* that is $m_{i}=\min\left\{\;j\;:(D_{j(i)}+1)\geq k\right\}$. *I* is the indicator function with value 1 when $P(M_{i}\leq m_{i}\;)<0.05$ and 0 when p ≥ 0.05. $P(M_{i}\leq m_{i}\;)$ is calculated as follows: $P\left(M_{i}\leq m_{i}\right)=1-P\left(M_{i}>\;m_{i}\right)=1-\sum_{s=0}^{k-1}e^{-\;U_{m_{i}\left(i\right)\;}\frac{C}{N}}{\left(U_{m_{i}\left(i\right)}\frac{C}{N}\right)}^{s}/s!.$

Under the null hypothesis, every individual person in a given region is equally likely to be a case, independent of other individuals and the location of residence. The null hypothesis of no clustering is rejected when the test statistic R is large. The method was applied as implemented in the R package SpatialEpi, version 1.2.3 [11]. For “nephroblastoma” scenario*, k* was set to 5 (for “all malignancies” to 50). These thresholds cover around the 75^th^ percentile of expected cases per district for the respective scenarios.

#### Spatial scan statistics

SSS in this study are represented by a modified approach adapted from Kulldorff [12]. SSS imposes a circular window on the map and lets the circle centroid move across the study region. For any given position of the centroid, the radius of the window is changed continuously between zero and an upper limit of radius or a maximum fraction of total population. Let *L_j(i)_* be the likelihood under the alternate hypothesis that there is a cluster in district *i* and its *j* closest neighbors, and let *L_0_* be the likelihood under the null hypothesis. It can then be shown that$\;\frac{L_{j(i)}}{L_{0}}={\left(\frac{D_{j(i)}}{U_{j(i)}\frac{C}{N}}\right)}^{D_{j(i)}}{\left(\frac{C-\;D_{j(i)}}{C-U_{j(i)}\frac{C}{N}}\right)}^{C-D_{j(i)}}.\;$As this likelihood ratio is maximized over all circles, it identifies the one that constitutes the most likely cluster. The test statistic is $T=max_{i,j}\frac{L_{j(i)}}{L_{0}}I\;\left(D_{j(i)}>{\frac{U_{j(i)}}{N}}^{\;}C\right)$ where I is the indicator function with value 1 when $D_{j(i)}>{\frac{U_{j(i)}}{N}}^{\;}C$ and 0 otherwise. The null hypothesis of no clustering is rejected when T is large. The method was applied as implemented in the R package SpatialEpi, version 1.2.3 [11]. The maximum population within the circles was set to 10 % of the total population.

#### Besag-York-Mollié method

In the Bayesian approach, the disease risk is estimated using a hierarchical model, comprising random effects that allow borrowing strength from the respective neighboring observations, therefore smoothing the spatial variation of relative risk and minimizing the likelihood of risk variation by chance. This makes the approach attractive for application in rare diseases and underpopulated areas. The general form is as follows (see e.g. [13,14]):

$$c_{i}\;|\;e_{i},\;RR_{i}\;\;∼\;\text{Poisson}\left(\lambda_{i}\right)\;\;\;\;\text{for}\;\;i=1,\ldots ,\;n\;$$

$$\lambda_{i}=\;e_{i}\;\times RR_{i}$$

$$\text{log}\left(RR_{i}\right)=\;\mu +m_{i}+\nu_{i},$$

where *RR_i_* is the relative risk in area *i*, which is modelled by an intercept term *μ,* an exchangeable area-specific effect $\nu_{i}$ and another spatially structured area-specific effect $m_{i}$. The spatially structured random effects can be estimated by a number of different models. Commonly, conditional autoregressive (CAR) prior distribution models are used in disease mapping studies. Spatial correlation between the random effects is defined by a binary *n x n* neighborhood matrix ***W***. In two neighboring districts denoted by *j∼i*, the random effects are correlated. Non-neighboring districts are modelled as being conditionally independent, given the remaining elements of m. The intrinsic autoregressive model includes the simplest CAR prior [15] and is referred to as the Besag, York and Mollié (BYM) model. The full conditional distribution in this model is then given by: $m_{i}|m_{-i},\;W,\;\tau_{l}^{2}\;∼\;N\left(\frac{1}{n_{i}}\sum_{j∼i}m_{j},\frac{\tau_{l}^{2}}{f_{i}}\right)$. The conditional expectation of $m_{i}$ is equal to the mean of the random effects in neighboring areas, while the conditional variance is inversely proportional to the number of neighbors $f_{i}$. Therefore, in the presence of strong spatial correlation, more neighbors yields increased information in the data about the value of the random effect. The parameter $\tau_{l}^{2}$ controls the variation between random effects.

While Inference in this such models is usually based on Markov chain Monte Carlo (MCMC) simulation, the presented approach applies the Integrated Nested Laplace Approximation (INLA) [16]. INLA has been shown to produce results comparable to MCMC sampling and is nowadays often used in spatial applications, see e.g. [17,18]. It was applied as implemented in the R package R-INLA, version 18.07.12 [19]. High-risk districts/ clusters were defined as regions where the estimated *RR* is larger than 1 as determined by its two-sided equal-tailed 95% credible interval. Minimally informative priors (1, 0.001) on the log-precisions of the unstructured and spatially structured effects (based on the log-gamma distribution; as is the default setting in R-INLA) were used.

### Performance of cluster detection methods

The performance of each of the various cluster detection methods and scenarios in this study is reported according to the quality criteria detailed below.

Variance estimates: Mean, standard deviation (SD) as well as lower and upper 95% confidence intervals (CI = mean ± 1.96 × SE) were calculated for all measured parameters (LCI and UCI).

## Ethics Statement

No humans and animals were directly involved in the study. Publicly available population based data was used from DESTATIS, GADM and the German Childhood Cancer Registry.

## Credit Author Statement

MSS and CStock: conceptualization, design of the study, coding, statistical analyses, drafting of manuscript; TL and MK: coding, CSpix: raw data supply; KB: conceptualization and drafting of manuscript; all authors contributed to revising the manuscript critically for important intellectual content and approved the final version.

## Declaration of Competing Interest

CStock is now full-time employee of Boehringer Ingelheim Pharma GmbH & Co. KG. The company had no role in design, analysis or interpretation of the present study. The remaining authors declare that they have no known competing financial interests or personal relationships which have or could be perceived to have influenced the work reported in this article.
